# Estimating Nonfatal Gunshot Injury Locations With Natural Language Processing and Machine Learning Models

**DOI:** 10.1001/jamanetworkopen.2020.20664

**Published:** 2020-10-14

**Authors:** Susan T. Parker

**Affiliations:** 1 University of Michigan School of Public Health, Ann Arbor, Michigan

## Abstract

**Importance:**

Nonfatal gunshot injuries are the most common firearm injury, but where they frequently occur remains unclear owing to data limitations. Natural language processing can be applied to medical text narratives of gunshot injury records to classify injury location and inform prevention efforts.

**Objective:**

To examine the performance of natural language processing (NLP) and machine learning models to predict nonfatal gunshot injury locations and generate new national estimates of the locations in which these injuries occur.

**Design, Setting, and Participants:**

Cross-sectional study of data from the National Electronic Injury Surveillance System Firearm Injury Surveillance Study on nonfatal gunshot injuries that occurred in the US between January 1, 1993, and December 31, 2015. The unweighted sample included 59 025 gunshot injuries that were initially treated at emergency departments. Data were analyzed from June 1, 2019 to July 24, 2020.

**Main Outcomes and Measures:**

The primary outcomes were classification of injury location and subsequent estimation of nonfatal gunshot injury location. The NLP was used to generate 6 sets of predictors, and 4 machine learning models were trained to classify the missing locations: multinomial support vector machines, lasso regression, XgBoost gradient descent, and feed-forward neural networks. For each of the 6 sets of NLP predictors, 70% of records with locations were randomly sampled to form the training set and the remaining 30% of records composed the test set. The best-performing model was validated by comparing the predicted locations were with those from existing national estimates of nonfatal gunshot injuries stratified by location and intent.

**Results:**

The unweighted sample included 59 025 nonfatal gunshot injuries; patients with these injuries were predominantly male (n = 52 630, [89.2%]), of Black race/ethnicity (n = 29 304 [49.6%]), and young (15-24 years; n = 27 037 [45.8%]). In total, 54 089 nonfatal gunshot injuries that were weighted to approximate national estimates were included in the analysis. Existing national estimates suggest that the most prevalent nonfatal gunshot injury location is the home (n = 14 764 [23.4%]), followed by the street or highway (n = 14 402 [22.9%]), and other public places (n = 7276 [11.6%]). After implementation of NLP classification, the most frequent gunshot injury location was the street or highway (n = 27 200 [46.1%]), followed by the home (n = 23 738 [37.7%]), and other public places (n = 10 439 [15.1%]).

**Conclusions and Relevance:**

The findings of this study suggest that NLP and machine learning models may be useful for classifying gunshot injury location and that most nonfatal gunshot injuries occur in the street or highway rather than in the home; these findings can inform future firearm injury prevention efforts.

## Key Points

**Question:**

Can natural language processing and machine learning methods be used to predict the locations of nonfatal shootings and improve the accuracy of existing national estimates of these gunshot injuries?

**Findings:**

This cross-sectional study of 22 years of data from the National Electronic Injury Surveillance System Firearm Injury Surveillance Study used natural language processing of unstructured medical text combined with machine learning models to classify the location of nonfatal gunshot injuries. Contrary to existing national estimates of these injuries that indicate they occur most often in homes, this analysis found that these injuries occur most often in the street or highway.

**Meaning:**

Natural language processing and machine learning may be used to predict gunshot injury locations, and this information could be used to improve the accuracy of existing national estimates of these locations and to inform future firearm injury prevention efforts.

## Teaser

This cross-sectional study uses data from the National Electronic Injury Surveillance System Firearm Injury Surveillance Study to test the ability of natural language processing combined with 4 machine learning models to predict the locations of nonfatal gunshot injuries.

## Introduction

Firearms are a leading cause of injury in the United States.^1^ Understanding where gunshot injuries occur is relevant for prevention, particularly prevention of nonfatal gunshot injuries, which occur 5 times more often than fatal gunshot injuries.^2^ However, information on the location of nonfatal firearm injury is frequently missing from national data sources. Localized studies of gunshot injuries^3,4,5,6^ vary in their assessment of the places where nonfatal injuries occur and prevention focus and are often limited owing to missing or imprecise data on the place where the injury occurred. Some studies have found that nonfatal gunshot injuries occur in a patient’s home and have suggested a focus on injury prevention in the home or in geographically proximate, concentrated areas.^3,4^ Other studies have used more precise location data and have found that gunshot injuries are less likely to occur in proximity to the patient’s residence, potentially owing to neighborhood factors and social ties.^5,6^ The limits of existing national data on nonfatal gunshot injuries have resulted in uncertainty about injury prevention efforts and the risks associated with firearms. Without accurate estimates of where nonfatal gunshot injuries most frequently occur, prevention efforts lack important data.

In this cross-sectional study, natural language processing (NLP) methods used unstructured medical text combined with machine learning models to predict the location where nonfatal gunshot injuries occur. Using unstructured medical text data from a national sample of nonfatal gunshot injuries, the location predictions were compared across different algorithms and NLP predictors. The predicted locations generated with NLP predictors and machine learning models were compared with existing locations from a national data source to reexamine the location of nonfatal gunshot injuries and intent patterns.

## Methods

The institutional review board at the University of Michigan deemed this study exempt from review and waived the requirement for participant informed consent because the data cannot be tracked to a human subject. This study followed the Strengthening the Reporting of Observational Studies in Epidemiology (STROBE) reporting guideline. Data were analyzed from June 1, 2019 to July 24, 2020.

Data used in this analysis were obtained from the National Electronic Injury Surveillance System (NEISS) Firearm Injury Surveillance Study (FISS), which included emergency department visits for gunshot injuries that occurred between January 1, 1993, and December 31, to 2015.^6^ The NEISS-FISS generates national estimates of nonfatal gunshot injuries from a probability sample of approximately 100 US emergency departments.^7^ The data are gathered and validated by medical coders through a Centers for Disease Control and Prevention partnership with the Consumer Product Safety Commission to supplement product safety injury surveillance with firearm injury surveillance. Although the location of injury is included in the NEISS-FISS data, almost half of the locations where gun injuries occur are not coded (eTable 1 in the Supplement). The NEISS-FISS also collects rich, unstructured medical narratives that document the circumstance of every gunshot injury.

### Study Sample

The study sample included all nonfatal gunshot injuries documented in NEISS-FISS that occurred between January 1, 1993, and December 31, to 2015. BB-gun injuries and injuries that are not gunshot wounds were excluded (n = 59 025). A study flow diagram documenting the sample is shown in eFigure 1 in the Supplement.

The NEISS-FISS received public attention for inaccurate estimates that suggested a misleading increase in nonfatal gunshot injuries.^8,9^ Researchers documented the sources of the problematic estimates, and found that the pattern originated from 2 sources.^10^ The first source was hospital replacement within the NEISS-FISS sample. Hospitals that handled few gunshot injuries were occasionally replaced with hospitals that handled a high volume of gunshot injuries. The second source was improvement in coding of injuries over time. Taken together, these changes suggested a false increase in national estimates of nonfatal gun injuries. To account for these issues, this analysis reports on national estimates informed by corrections suggested in Cook et al.^10^ Three influential, high-volume hospital replacements were imputed to prior year levels in this analysis (eTable 2 in the Supplement).

### Nonfatal Gunshot Injury Location Classification 

The NEISS-FISS sample contains 9 labeled location categories, but some categories were reclassified to improve their generalizability for injury prevention and prediction. Location categories, such as a gunshot injury that occurred on a farm or the difference between an apartment and a house, were not feasible to predict because these categories each represent less than 1% of the injury data locations (see eTable 1 in the Supplement). In this analysis, nonfatal gunshot injuries were therefore classified as occurring in the following locations: (1) in a home or apartment, (2) in the street or highway, (3) other public place or (4) other. Other public place is an existing injury location code that was unaltered; it refers to injuries that occurred in public places such as stores and restaurants. Remaining injury location codes in the NEISS-FISS sample include recreation, industry, and school, and each of these categories comprise less than 2% of the nonfatal gunshot injuries (see eTable 1 in the Supplement). These cases were combined and recoded as *Other* in the predictive models.

### Natural Language Processing Predictors

Natural language processing of NEISS-FISS unstructured medical text was used to generate predictors to classify locations of nonfatal gunshot injuries. Each gunshot injury included in the NEISS-FISS contains a narrative describing the injury circumstances (eg, sex and age of the individual, primary body part affected, place where the injury occurred, and whether the individual was sitting or standing) compiled by trained medical coders.

Unique words that appeared in the medical narratives were identified and used to form predictors to indicate whether the word, or a combination of words, appeared in the narrative. To ensure that results were not driven by differences in medical text among records with missing vs nonmissing location data, the word predictors were applied to different samples and NLP modeling strategies. First, to assess the robustness of classification to selection in the test and training sets, 2 sets of NLP predictors were constructed using (1) shared words (n = 9528) in all records with missing and nonmissing location data and (2) all words (n = 15 358) in any record. Next, for each of the 2 sets of words, NLP predictors were generated to reduce dimensionality and generate meaningful features (eg, nightclub, arrest, intoxication, domestic). Term frequency-inverse document frequency (TF-IDF) weighting was used to weight the frequency of each individual word in the comments so that words that occurred frequently, such as *shot*, were downweighted vs words such as *cleaning*, which would provide additional information about the injury circumstances. Second, n-grams were used to create sequential word pairings as features with information about word sequencing. In addition, word embeddings (ie, a set of language modeling and feature learning techniques in NLP) was used to capture semantic relationships between words via the Global Vectors for Word Representation algorithm.^11^ Words that appeared less than 10 times or in less than 1% of the records were omitted.

### Machine Learning Classification and Evaluation

Six sets of NLP features were used to train and evaluate 4 classification models. Classification models were selected for their suitability to avoid overfitting when selection existed between training and test sets and for regularization of wide feature sets. With sufficient training data, algorithms that are local learners, rather than global, can generate unbiased classification results even if the training data are subject to selection.^12,13^ Specifically, multinomial support vector machines (SVMs) and Lasso regression were selected for this reason along with XgBoost gradient descent and feed-forward neural networks.^14^

Model performance was assessed across 6 different NLP predictor constructions that varied both by word content and feature construction to ascertain whether the content, similarity, sequence, or frequency of predictors mattered for performance. For each of the 6 sets of NLP predictors, 70% of records with locations were randomly sampled to form the training set and the remaining 30% of records composed the test set. 5-fold cross-validation was used to fit each multinomial classification model to prevent overfitting. Predictions from each fitted model were applied to the test set to evaluate out-of-sample predictions by accuracy, precision, recall, and the area under the curve (AUC) of out-of-sample test set predictions by each location. Misclassification by location for the preferred each classifier model is reported described in eTable 5 in the Supplement. The preferred fitted model was applied to missing location data to classify where gunshot injuries occurred.

### Statistical Analysis

Descriptive statistics were used to compare missing vs nonmissing location data with respect to the severity and location of the injury, the demographic characteristics of the injured patients, the intent of the shooting, hospital, and other variables. Training, test, and missing location data were also compared. The words present in the narratives of records with missing and nonmissing location data were compared using 2-tailed, Spearman correlations of ranked word frequency to ascertain the magnitude of the differences between unstructured text in records with missing and nonmissing location data.^15^

Location predictions from the best-performing model were combined with NEISS-FISS sample case weights and existing records with nonmissing location data to generate national estimates of the locations of gunshot injuries. To account for increases in gunshot injuries in the national estimates that were attributable to hospital replacement, 3 probability sampling units with large replacement volumes were imputed to their prior year distribution in the most influential hospital sample replacements (eTable 2 in the Supplement). National estimates generated with predicted locations were compared with estimates without predicted locations or with the existing NEISS-FISS gunshot injury location estimates, using 2-sided χ^2^ tests. Using these combined locations from the best-performing model’s classification results, 95% CIs were bootstrapped using 500 replications of the preferred model predictions and the NEISS direct variance estimation procedures.^16^ Predicted locations were used to examine intent and the patient’s race and sex by location of injury.

Analyses were performed using R, version 3.4.3 (R Foundation for Statistical Computing) with the text2vec, glmnet, e1071 and nnet packages.^17,18^ The threshold for statistical significance was an an α level of .05.

## Results

Table 1 presents the characteristics of patients with nonfatal gunshot injuries between January 1, 1993, to December 31, 2015, as well as details about the affected body part, circumstances of the injury, and hospital attributes. The unweighted sample comprises 59 025 nonfatal gunshot injuries, of which victims were predominantly male (n = 52 630, [89.2%]), of Black race/ethnicity (n = 29 304 [49.6%]), and young (15-24 years; n = 27 037 [45.8%]), and the incident intent was assault (42 099 [71.3%]). Patients whose records were missing injury location data often were hospitalized, wounded in the upper trunk, and shot during an assault. The records with missing and nonmissing location data had different distributions across variables, including whether the injury occurred during a crime, the primary body part affected, and the victim’s age, but the records differ less by hospital stratum (eTable 3 in the Supplement). However, the NLP predictors that were shared between records with missing and nonmissing location data were correlated. Among the top 200 words in records with missing and nonmissing location data, 4 words were present in records with missing location and were not present in those with location data (eFigure 2 in the Supplement). The Spearman correlation between ranked words in each set of NLP predictors for the top 200 words was 0.92 (*P* < .001) and did not differ with rarer word occurrences (eTable 4 in the Supplement).

**Table 1.** Patient Demographic Characteristics, Injury Type, Location, and Intent

Characteristic	No. (%)			
	Missing (n = 26 392)	Test set (n = 9790)	Train set (n = 22 843)	Overall (n = 59 025)
Patient sex^a^				
Male	23 915 (90.6)	8615 (88.0)	20 100 (88.0)	52 630 (89.2)
Female	2468 (9.4)	1173 (12.0)	2737 (12.0)	6378 (10.8)
Patient disposition				
Treated/released	10 240 (38.8)	4166 (42.6)	9823 (43.0)	24 229 (41.0)
Transferred/released	753 (2.9)	368 (3.8)	829 (3.6)	1950 (3.3)
Transferred/hospital	32 (0.1)	11 (0.1)	27 (0.1)	70 (0.1)
Hospitalized	14 944 (56.6)	5063 (51.7)	11 709 (51.3)	31 716 (53.7)
Observation	198 (0.8)	145 (1.5)	331 (1.4)	674 (1.1)
Crime involved in injury				
Unknown	19 368 (73.4)	5170 (52.8)	12 110 (53.0)	36 648 (62.1)
Yes	4178 (15.8)	1975 (20.2)	4622 (20.2)	10 775 (18.3)
No	2846 (10.8)	2645 (27.0)	6111 (26.8)	11 602 (19.7)
Race of patient				
Not stated	5992 (22.7)	1621 (16.6)	3740 (16.4)	11 353 (19.2)
White	4284 (16.2)	2229 (22.8)	5129 (22.5)	11 642 (19.7)
Black	12 983 (49.2)	4890 (49.9)	11 431 (50.0)	29 304 (49.6)
Other	3133 (11.9)	1050 (10.7)	2543 (11.1)	6726 (11.4)
Age group levels, y				
0-14	755 (2.9)	485 (5.0)	1037 (4.5)	2277 (3.9)
15-24	12 576 (47.7)	4259 (43.5)	10 202 (44.7)	27 037 (45.8)
24-34	7205 (27.3)	2624 (26.8)	5876 (25.7)	15 705 (26.6)
35-44	3375 (12.8)	1179 (12.0)	2898 (12.7)	7452 (12.6)
45-54	1414 (5.4)	702 (7.2)	1519 (6.6)	3635 (6.2)
55-65	575 (2.2)	267 (2.7)	682 (3.0)	1524 (2.6)
>65	328 (1.2)	221 (2.3)	524 (2.3)	1073 (1.8)
Primary body part affected^a^				
Head/neck	3593 (13.6)	1449 (14.8)	3368 (14.7)	8410 (14.2)
Trunk				
Upper	5399 (20.5)	1589 (16.2)	3714 (16.3)	10 702 (18.1)
Lower	4601 (17.4)	1562 (16.0)	3578 (15.7)	9741 (16.5)
Arm/hand	3722 (14.1)	1571 (16.0)	3759 (16.5)	9052 (15.3)
Leg/foot	8700 (33.0)	3465 (35.4)	8063 (35.3)	20 228 (34.3)
Other	258 (1.0)	95 (1.0)	225 (1.0)	578 (1.0)
Stratum of hospital				
Small	603 (2.3)	536 (5.5)	1235 (5.4)	2374 (4.0)
Medium	1599 (6.1)	636 (6.5)	1627 (7.1)	3862 (6.5)
Large	3749 (14.2)	1429 (14.6)	3357 (14.7)	8535 (14.5)
Very large	19 728 (74.7)	6796 (69.4)	15 790 (69.1)	42 314 (71.7)
Children’s	713 (2.7)	393 (4.0)	834 (3.7)	1940 (3.3)
Incident intent^a^				
Unknown	4678 (17.7)	479 (4.9)	1239 (5.4)	6396 (10.8)
Unintentional	2602 (9.9)	1490 (15.2)	3342 (14.6)	7434 (12.6)
Assault	17 951 (68.0)	7261 (74.2)	16 887 (73.9)	42 099 (71.3)
Suicide	930 (3.5)	453 (4.6)	1127 (4.9)	2510 (4.3)

^a^ Unknown patient categories are omitted if they comprise less than 2% of the patient characteristic; therefore, the numbers may not sum to the total number listed in the heading.

Table 2 describes the performance of each multinomial classification algorithm by NLP predictor set. Among classifiers (ie, home, street, public place, other) fitted on the set of all word NLP predictors, models fitted on TF-IDF predictors had the most accurate performance, specifically Lasso regression with all words and TF-IDF normalization (accuracy, 0.783) and SVM with TF-IDF normalization (accuracy, 0.747). Among classifiers fitted on the set of shared word NLP predictors, Lasso regression with TF-IDF (accuracy, 0.787) and SVM with TF-IDF (accuracy, 0.772) were most accurate both overall and among local classifiers. The accuracy loss between models fitted on the set of all words compared with shared words was 0.4% for Lasso regression and 2.5% for SVM models. Among all models, TF-IDF normalization and n-grams were more often associated with more accurate model performance rather than word embeddings. The shared words Lasso regression model with TF-IDF normalization correctly classified locations most frequently (81.9% of home locations, 72.9% of street or highway, 72.6% of public place gunshot injuries, and 73.2% of other locations). Misclassifications for this model were most often instances of incorrect classification of gunshot injuries that occurred in the street as other public place, in which 12.3% of street locations were misclassified as public place and 18.9% of public place locations were misclassified as street (eTable 5 in the Supplement). Predicted missing location data by each model and NLP predictor set are presented in eTable 6 in the Supplement. In the most accurate Lasso regression model trained on shared words, 30.0% of the missing location data were classified as having occurred in a home, 56.8% in the street or highway, 12.4% in public places and 0.8% in other locations.

**Table 2.** Model Performance by Classifier

Model	Precision				Recall				Accuracy	AUC
	Home	Street	Public Place	Other	Home	Street	Public Place	Other		
Lasso shared words										
TF-IDF normalization	0.819	0.792	0.726	0.732	0.815	0.876	0.639	0.478	0.787	0.805
N-grams	0.769	0.756	0.641	0.532	0.770	0.848	0.569	0.212	0.734	0.740
Word embeddings	0.700	0.707	0.611	0.437	0.720	0.819	0.489	0.133	0.685	0.679
Lasso all words										
TF-IDF normalization	0.818	0.789	0.715	0.730	0.812	0.875	0.630	0.458	0.783	0.805
N-grams	0.771	0.755	0.631	0.527	0.769	0.850	0.562	0.197	0.732	0.737
Word embeddings	0.711	0.700	0.593	0.473	0.726	0.818	0.472	0.126	0.682	0.680
SVM shared words										
TF-IDF normalization	0.778	0.781	0.740	0.740	0.810	0.869	0.599	0.442	0.772	0.766
N-grams	0.757	0.776	0.675	0.609	0.799	0.852	0.588	0.179	0.748	0.734
Word embeddings	0.739	0.749	0.684	0.605	0.769	0.858	0.553	0.173	0.733	0.697
SVM all words										
TF-IDF normalization	0.761	0.755	0.710	0.615	0.791	0.858	0.580	0.204	0.747	0.716
N-grams	0.758	0.767	0.671	0.566	0.788	0.857	0.577	0.164	0.743	0.726
Word embeddings	0.744	0.740	0.661	0.609	0.766	0.862	0.527	0.148	0.726	0.692
NN shared words										
TF-IDF normalization	0.626	0.656	0.607	0.436	0.685	0.816	0.343	0.080	0.637	0.621
N-grams	0.765	0.765	0.627	0.565	0.777	0.831	0.593	0.182	0.734	0.742
Word embeddings	0.709	0.716	0.607	0.467	0.718	0.823	0.509	0.142	0.690	0.693
NN all words										
TF-IDF normalization	0.771	0.758	0.650	0.548	0.778	0.841	0.592	0.188	0.738	0.742
N-grams	0.767	0.760	0.616	0.587	0.769	0.838	0.581	0.166	0.730	0.737
Word embeddings	0.704	0.700	0.594	0.434	0.733	0.817	0.456	0.120	0.680	0.688
XgBoost shared words										
TF-IDF normalization	0.787	0.613	0.831	0.677	0.575	0.949	0.381	0.493	0.680	0.849
N-grams	0.765	0.602	0.794	0.576	0.581	0.951	0.332	0.228	0.661	0.817
Word embeddings	0.626	0.656	0.607	0.436	0.685	0.816	0.343	0.080	0.637	0.695
XgBoost all words										
TF-IDF normalization	0.763	0.610	0.824	0.567	0.584	0.947	0.362	0.279	0.669	0.816
N-grams	0.766	0.603	0.793	0.533	0.579	0.952	0.332	0.235	0.660	0.813
Word embeddings	0.630	0.647	0.580	0.448	0.682	0.830	0.296	0.086	0.632	0.703

Abbreviations: NN, neural network; TF-IDF, term frequency-inverse document frequency; SVM, support vector machines.

**Figure 1.**
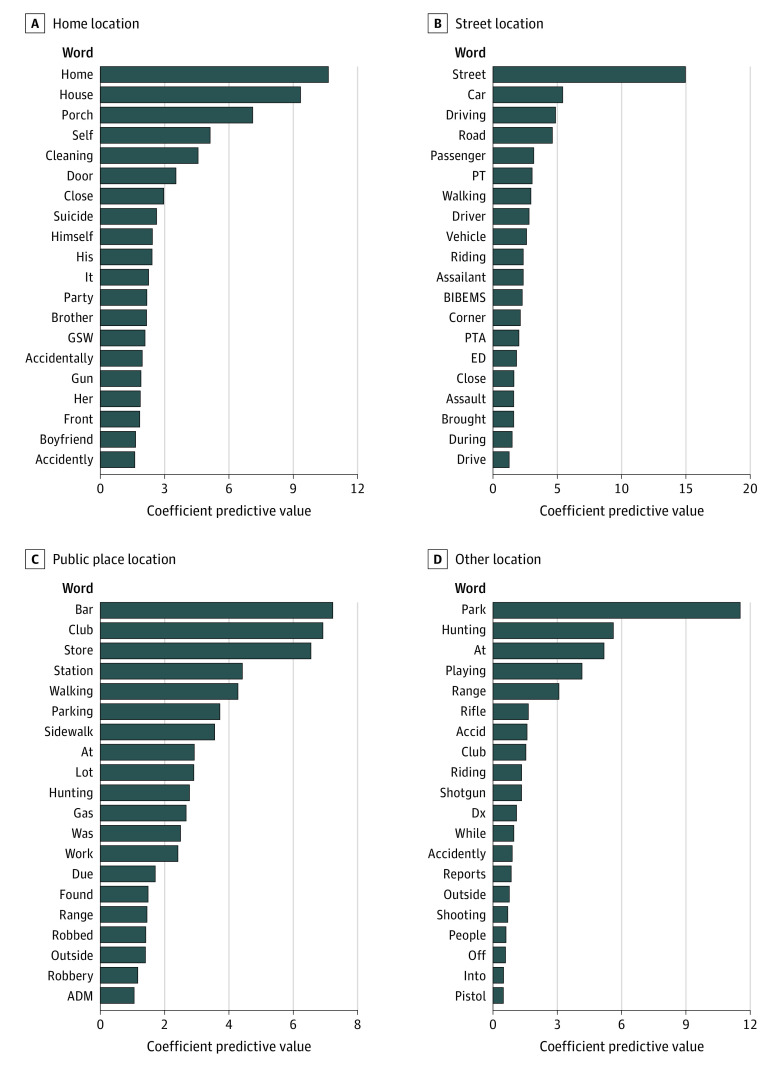
Predictive Value of Specific Words From the Narrative Text Stratified by Gunshot Injury Location Figure shows the predictive words and medical coder abbreviations from the Lasso regression model fitted on shared words with term frequency-inverse document frequency normalization. Some duplication was present because of words used by the coders (ie, “accidently” and “accidentally”).

Figure 1 plots predictive words from the Lasso regression model fitted on shared words with TF-IDF normalization that are influential in predicting the missing locations. Words are plotted by the magnitude of their fitted coefficient. For nonfatal gunshot injuries that occur in a home, terms reflecting relationships, such as *husband* and *boyfriend*, as well as self-harm are important predictors. Nonfatal gunshot injuries that occurred in public places were best predicted by terms including *store*, *bar*, and *club*. For nonfatal gunshot injury locations in streets or highways, words that reflect this location, such as *street*, *car*, and *driving* are predictive and terms such as *assault* are predictive of the incident intent.

**Figure 2.**
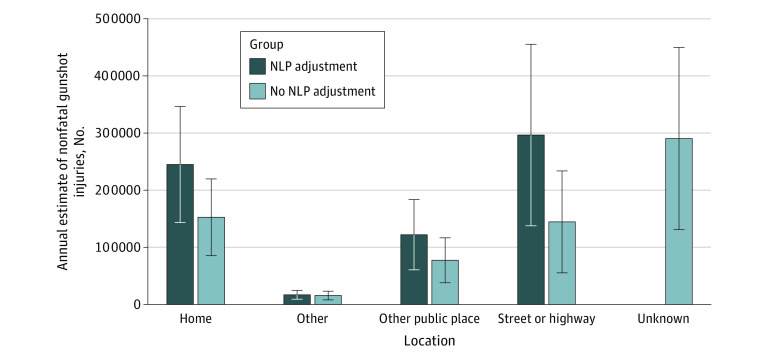
Weighted Annual National Estimates of Nonfatal Gunshot Injury Locations With and Without Natural Language Processing Adjustment Stratified by Location Error bars indicate the 95% CIs.

Figure 2 shows weighted national estimates of nonfatal gunshot injury locations with NLP and without NLP predicted locations. Without classification of the records with missing location data, the largest category of weighted nonfatal gun injury locations was unknown (mean annual estimate, 29 029 [39.5%]; 95% CI, 13 095.0-44 961.0), followed by injuries in the home (mean annual estimate, 15 257 [23.4%]; 95% CI, 8543.9-21 969.5), injuries in the street or highway (mean annual estimate, 14 447 [22.9%]; 95% CI, 5525.4-23 368.9), and injuries in other public places (mean annual estimate, 7732 [11.6%]; 95% CI, 3801.9-11 662.8) (eTable 7 in the Supplement). With NLP and classification, street or highway was the most frequent gun injury location (mean annual estimate, 27 200 [43.6%]; 95% CI, 13 769.6-45 517.5), followed by injuries in the home (mean annual estimate, 23 738 [36.0%]; 95% CI, 14 335.2-34 656.5), injuries in other public places (mean annual estimate, 10 439 [15.1%]; 95% CI, 6061.7-18 337.5).

Table 3 reports weighted national estimates of nonfatal gunshot injury locations stratified by intent and victim sex and race. Within each location, the distributions with predicted locations were similar to those without predicted locations. Assault and unintentional injury remain fairly equally associated with injury in the home and victim race and sex also remain stable. For each location, victims were most likely to be Black individuals and to have endured assault.

**Table 3.** Intent and Victim Race and Sex by Location

Location/Variable	Weighted mean annual estimate, No. (%)	
	Unadjusted NEISS-FISS	NLP-Adjusted NEISS-FISS
**Home**		
Intent		
Assault	6066.9 (40.5)	9841.3 (39.9)
Self-harm	2065.3 (13.8)	3331.7 (13.5)
Unintentional	5846.8 (39.1)	8888.9 (36)
Unknown	988.7 (6.6)	2606.6 (10.6)
Victim race		
Black	6288.0 (47)	10 670.3 (42.7)
Not stated	4284.9 (15.5)	4315.7 (17.3)
Other	3419.7 (14.1)	3560.4 (14.3)
White	10 540.9 (23.4)	6423.8 (25.7)
Victim sex		
Female	2553.7 (16.9)	3828.9 (15.4)
Male	12 531.9 (83.1)	21 007.0 (84.6)
**Street/highway**		
Intent		
Assault	13 170.2 (92.5)	25 427.1 (85.6)
Self-harm	101.2 (0.7)	172.4 (0.6)
Unintentional	388.6 (2.7)	792.7 (2.7)
Unknown	580.1 (4.1)	3327.0 (11.2)
Victim race		
Black	8566.8 (59.3)	16 267.8 (55.2)
Not stated	1655.3 (11.5)	4335.7 (30.0)
Other	2352.3 (16.3)	5002.7 (34.6)
White	1872.9 (13.0)	3840.9 (26.6)
Victim sex		
Male	1219.9 (8.1)	2440.0 (8.4)
Female	13 221.3 (91.9)	27 665.3 (91.6)
**Other public place**		
Intent		
Assault	6032.1 (79.8)	8756.5 (78.7)
Self-harm	133.3 (1.8)	147.3 (1.3)
Unintentional	1022.7 (13.5)	1347.9 (12.1)
Unknown	366.4 (4.9)	874.1 (7.9)
Victim race		
Black	2909.1 (37.6)	4802.3 (38.9)
Not stated	1202.8 (15.6)	2152.1 (17.4)
Other	1582.0 (20.5)	2446.7 (19.8)
White	2038.5 (26.4)	2951.8 (23.9)
Victim sex		
Female	847.6 (11.0)	1160.4 (10.2)
Male	6884.7 (89.8)	10 202.5 (89.8)

Abbreviations: NEISS-FISS, National Electronic Injury Surveillance System Firearm Injury Surveillance System; NLP, natural language processing.

## Discussion

This cross-sectional study used NLP (to generate predictors from unstructured medical narrative text) and machine learning models to accurately classify nonfatal gunshot injury locations to home, street or highway, public place, or other categories. Model performance did not appear to be influenced by selection among records with missing and non-missing location data when comparing NLP predictors and models across different specifications. Contrary to national estimates without predicted locations, in this study, nonfatal gunshot injuries occurred most frequently in the street or highway rather than in homes. Among nonfatal gunshot injuries that occur in the home, predicted locations suggested that, in terms of incident intent, assault is as likely as unintentional injury.

### Limitations

This study has several limitations. Although the machine learning models reduced misleading missing location data from nonfatal gunshot injury records, they do not correct for large SEs from the relatively small NEISS-FISS hospital sample. Caution should be used in interpreting NEISS-FISS patterns over time because of imprecise estimates that have been shown to be sensitive to hospital sample inclusion. Efforts to account for these sample problems have been implemented by adjusting the national estimates presented. Second, selection in the training data could be a source of bias in predicted locations. Efforts to minimize selection in predicted locations included the use of machine learning models that were robust to selection and the use of features independent of information present in only the missing or non-missing training set. The existing ordering without predicted locations of where nonfatal gunshot injuries occurred was not preserved in any model; however, it is possible that classification or misclassification of location was still associated with hospital attributes that result in the missing location codes. In addition, not all records with missing location data could be verified using only NEISS-FISS medical text.

## Conclusions

Contrary to existing national estimates of where nonfatal gunshot injuries most frequently occur, the findings of this cross-sectional study, which used NLP and machine learning models to predict these locations, suggest that nonfatal gunshot injuries occur more often in the street or highway rather than in homes. Where a nonfatal gunshot injury occurs helps to inform firearm injury prevention efforts, and the medical narratives included in the records from the NEISS-FISS offer valuable insight into injury location when combined with natural language processing techniques.

## Notes

Supplement. **eFigure 1.** Study Flow Diagram **eTable 1.** Nonfatal Gun Injury Location Categories and Recoded Prediction Category **eTable 2.** Hospital Probability Sampling Unit Estimate Imputations **eTable 3.** Wounded Individual Characteristics, Circumstances, Injury and Disposition by Missing and Non-Missing Location Data **eFigure 2.** Comparison of NLP Predictors Rank by Missing and Non-Missing Location Data **eTable 4.** Spearman Correlation between NLP Predictors Rank by Missing and Non-Missing Location Data **eTable 5.** Misclassification Errors by Best-performing Model **eTable 6.** Predicted Missing Gun Injury Locations by Model Type **eTable 7.** Comparison of National Estimates of Non-Fatal Shootings with NLP
