# Supplementary material for: Estimating Nonfatal Gunshot Injury Locations With Natural Language Processing and Machine Learning Models
Source: JAMA Netw Open. 2020 Oct 14;3(10):e2020664. doi: 10.1001/jamanetworkopen.2020.20664 (PMC7557517; doi:10.1001/jamanetworkopen.2020.20664)
Supplement: Supplement. — eFigure 1. Study Flow Diagram eTable 1. Nonfatal Gun Injury Location Categories and Recoded Prediction Category eTable 2. Hospital Probability Sampling Unit Estimate Imputations eTable 3. Wounded Individual Characteristics, Circumstances, Injury and Disposition by Missing and Non-Missing Location Data eFigure 2. Comparison of NLP Predictors Rank by Missing and Non-Missing Location Data eTable 4. Spearman Correlation between NLP Predictors Rank by Missing and Non-Missing Location Data eTable 5. Misclassification Errors by Best-performing Model eTable 6. Predicted Missing Gun Injury Locations by Model Type eTable 7. Comparison of National Estimates of Non-Fatal Shootings with NLP [file jamanetwopen-e2020664-s001.pdf]

Supplemental Online Content

Parker ST. Estimating nonfatal gunshot injury locations with natural language processing and machine learning models. *JAMA Netw Open*. 2020;3(10):e2020664. doi:10.1001/jamanetworkopen.2020.20664

eFigure 1. Study Flow Diagram
eTable 1. Nonfatal Gun Injury Location Categories and Recoded Prediction Category
eTable 2. Hospital Probability Sampling Unit Estimate Imputations
eTable 3. Wounded Individual Characteristics, Circumstances, Injury and Disposition by Missing and Non-Missing Location Data
eFigure 2. Comparison of NLP Predictors Rank by Missing and Non-Missing Location Data
eTable 4. Spearman Correlation between NLP Predictors Rank by Missing and Non-Missing Location Data
eTable 5. Misclassification Errors by Best-performing Model
eTable 6. Predicted Missing Gun Injury Locations by Model Type
eTable 7. Comparison of National Estimates of Non-Fatal Shootings with NLP

This supplemental material has been provided by the authors to give readers additional information about their work.

eFigure 1. Study Flow Diagram.

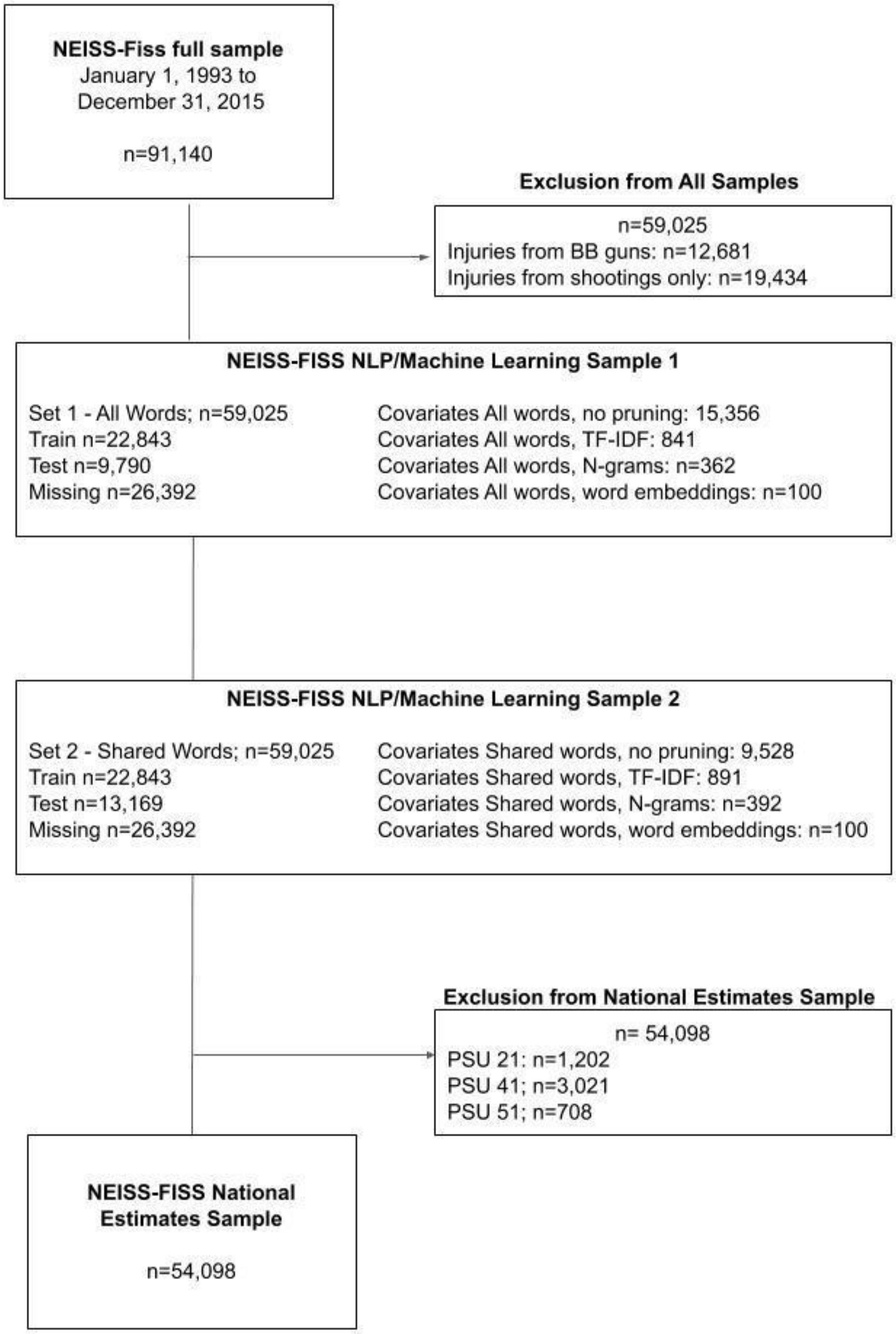

© 2020 Parker ST. *JAMA Network Open.*

eTable1. Nonfatal Gun Injury Location Categories and Recoded Prediction Category

**NEISS-FISS Location Code**	**Overall (N=78459)**	**Prediction Category**
Unknown*	34563 (44.1%)	Not applicable
Home	14492 (18.5%)	Home
Farm	114 (0.1%)	Other
Apartment or Condo	71 (0.1%)	Home
Street or Highway	17613 (22.4%)	Street or Highway
Other Public Place	9732 (12.4%)	Other Public Place
Mobile Home	112 (0.1%)	Home
Industry	54 (0.1%)	Other Public Place
School	286 (0.4%)	Other Public Place
Recreation	1422 (1.8%)	Other Public Place

Note: Unknown locations are referred to as missing location data. Fitted models are applied to records with this location code in order to predict a location for this set of shooting injury records.

© 2020 Parker ST. *JAMA Network Open.*

eTable 2. Hospital Probability Sampling Unit Estimate Imputations

	PSU - Unadjusted			PSU- Adjusted		
Year	PSU 21	PSU 41	PSU 51	PSU 21	PSU 41	PSU 51
1993	10	11	111	10	11	111
1994	7	16	77	7	16	77
1995	14	5	35	14	5	35
1996	8	7	29	8	7	29
1997	3	1	28	3	1	28
1998	4	4	34	4	4	34
1999	2	4	39	2	4	39
2000	6	2	27	6	2	27
2001	2	5	22	2	5	22
2002	4	2	25	4	2	25
2003	0	4	13	4	4	13
2004	29	2	16	4	2	16
2005	99	4	18	4	4	18
2006	125	6	22	4	6	22
2007	149	1	7	4	1	22
2008	139	1	149	4	1	22
2009	125	0	62	4	0	22
2010	123	3	118	4	3	22
2011	106	0	122	4	3	22
2012	91	358	138	4	3	22
2013	94	796	95	4	3	22
2014	78	888	113	4	3	22
2015	96	994	102	4	3	22
Total	1314	3114	1402	112	93	694

© 2020 Parker ST. *JAMA Network Open.*

eTable 3. Wounded Individual Characteristics, Circumstances, Injury and Disposition by Missing and Non-Missing Location Data

	Missing (N=32,633)	Non-Missing (N=26,392)	Overall (N=59,025)	P-Value
**Sex**				
Male	28715 (88.0%)	23915 (90.6%)	52630 (89.2%)	p<.001
Female	3910 (12.0%)	2468 (9.4%)	6378 (10.8%)	
**Disposition**				
(1) Treated/released	13989 (42.9%)	10240 (38.8%)	24229 (41.0%)	p<.001
(2) Transfd/released	1197 (3.7%)	753 (2.9%)	1950 (3.3%)	
(3) Transfd/hospital	38 (0.1%)	32 (0.1%)	70 (0.1%)	
(4) Hospitalized	16772 (51.4%)	14944 (56.6%)	31716 (53.7%)	
(5) Observation	476 (1.5%)	198 (0.8%)	674 (1.1%)	
(6) AMA/LWBS	104 (0.3%)	141 (0.5%)	245 (0.4%)	
(9) Unknown	57 (0.2%)	84 (0.3%)	141 (0.2%)	
**Crime**				
Unknown	17280 (53.0%)	19368 (73.4%)	36648 (62.1%)	p<.001
Yes	6597 (20.2%)	4178 (15.8%)	10775 (18.3%)	
No	8756 (26.8%)	2846 (10.8%)	11602 (19.7%)	
**Race**				
Not Stated	5361 (16.4%)	5992 (22.7%)	11353 (19.2%)	p<.001
White	7358 (22.5%)	4284 (16.2%)	11642 (19.7%)	
Black	16321 (50.0%)	12983 (49.2%)	29304 (49.6%)	
Other	3593 (11.0%)	3133 (11.9%)	6726 (11.4%)	
**Age**				
(0) Unknown	158 (0.5%)	164 (0.6%)	322 (0.5%)	p<.001
(1) 0-14	1522 (4.7%)	755 (2.9%)	2277 (3.9%)	
(2) 15-24	14461 (44.3%)	12576 (47.7%)	27037 (45.8%)	
(3) 25-34	8500 (26.0%)	7205 (27.3%)	15705 (26.6%)	
(4) 35-44	4077 (12.5%)	3375 (12.8%)	7452 (12.6%)	
(5) 45-54	2221 (6.8%)	1414 (5.4%)	3635 (6.2%)	

© 2020 Parker ST. *JAMA Network Open.*

(6) 55-64	949 (2.9%)	575 (2.2%)	1524 (2.6%)	
(7) 65+	745 (2.3%)	328 (1.2%)	1073 (1.8%)	
**Body Part**				
Unknown	195 (0.6%)	119 (0.5%)	314 (0.5%)	p<.001
Head/neck	4817 (14.8%)	3593 (13.6%)	8410 (14.2%)	
Upper Trunk	5303 (16.3%)	5399 (20.5%)	10702 (18.1%)	
Lower Trunk	5140 (15.8%)	4601 (17.4%)	9741 (16.5%)	
Arm/hand	5330 (16.3%)	3722 (14.1%)	9052 (15.3%)	
Leg/foot	11528 (35.3%)	8700 (33.0%)	20228 (34.3%)	
Other	320 (1.0%)	258 (1.0%)	578 (1.0%)	
**Hospital Stratum**				
Small	1771 (5.4%)	603 (2.3%)	2374 (4.0%)	p<.001
Medium	2263 (6.9%)	1599 (6.1%)	3862 (6.5%)	
Large	4786 (14.7%)	3749 (14.2%)	8535 (14.5%)	
Very Large	22586 (69.2%)	19728 (74.7%)	42314 (71.7%)	
Childrens	1227 (3.8%)	713 (2.7%)	1940 (3.3%)	
**Intent**				
Unknown	1718 (5.3%)	4678 (17.7%)	6396 (10.8%)	p<.001
Unintentional	4832 (14.8%)	2602 (9.9%)	7434 (12.6%)	
Assault	24148 (74.0%)	17951 (68.0%)	42099 (71.3%)	
Suicide	1580 (4.8%)	930 (3.5%)	2510 (4.3%)	

© 2020 Parker ST. *JAMA Network Open.*

eFigure 2. Comparison of NLP Predictors by Missing and Non-Missing Location Data

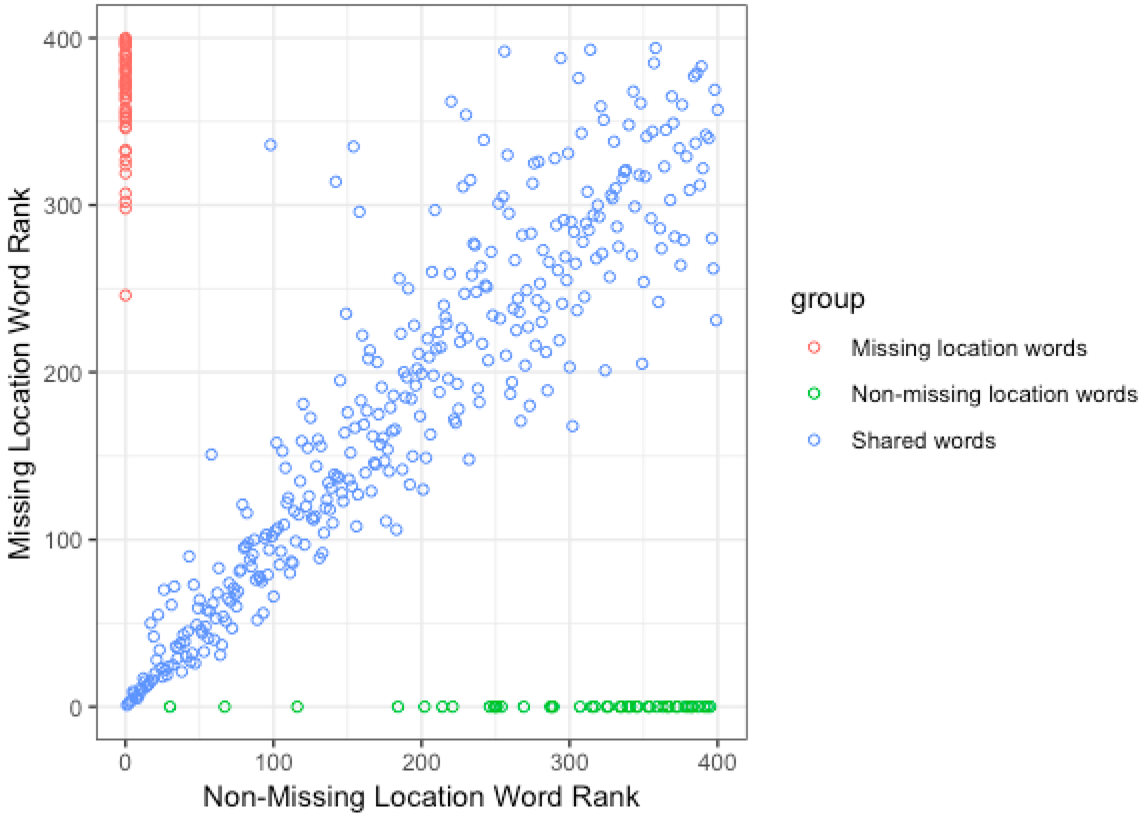

© 2020 Parker ST. *JAMA Network Open.*

eTable 4. Spearman Correlation between NLP Predictors Rank by Missing and Non-Missing Location Data

NLP Predictor Frequency Rank	Spearman Correlation	p-value
100	0.84	p<.001
200	0.92	p<.001
400	0.92	p<.001
600	0.91	p<.001
781	0.92	p<.001

© 2020 Parker ST. *JAMA Network Open.*

eTable 5. Misclassification Errors by Preferred Model (Lasso TF-IDF NLP Shared Word Model)

Predicted Class	True Class				
	Home	Street	Public Place	Other	Total
Home	79.50%	8.34%	9.77%	2.40%	100.00%
Street	9.98%	76.56%	12.24%	1.22%	100.00%
Public Place	11.48%	18.85%	61.18%	8.49%	100.00%
Other	10.17%	0.00%	18.64%	71.19%	100.00%

© 2020 Parker ST. *JAMA Network Open.*

eTable 6. Predicted Missing Locations by Model Type

Classifier	Home	Street	Public Place	Other Location
Lasso: All words, TF-IDF normalization	29.66	56.53	13.11	0.71
Lasso: All words, n-grams	29.90	54.99	14.40	0.71
Lasso: All words, embeddings	32.91	55.60	10.82	0.67
Lasso: Shared words, TF-IDF normalization	30.04	56.76	12.43	0.77
Lasso: Shared words, n-grams	30.01	55.48	13.81	0.71
Lasso: Shared words, embeddings	34.09	55.35	10.05	0.52
SVM: All words, TF-IDF normalization	34.55	53.97	11.07	0.41
SVM: All words, n-grams	34.50	51.45	13.60	0.45
SVM: All words, embeddings	31.07	57.54	10.98	0.41
SVM: Shared words, TF-IDF normalization	33.08	55.57	10.91	0.45
SVM: Shared words, n-grams	36.74	49.69	13.13	0.44
SVM: Shared words, embeddings	32.07	56.58	10.91	0.43
NN: All words, TF-IDF normalization	29.72	55.38	14.16	0.73
NN: All words, n-grams	30.64	52.73	16.07	0.56
NN: All words, embeddings	30.64	52.73	16.07	0.56
NN: Shared words, TF-IDF normalization	36.01	57.12	6.23	0.64
NN: Shared words, n-grams	31.07	52.71	15.58	0.65
NN: Shared words, embeddings	32.02	56.13	11.06	0.78
XgBoost: All words, TF-IDF normalization	12.14	85.31	1.74	0.81
XgBoost: All words, n-grams	11.20	86.13	2.07	0.60
NN: All words, embeddings	34.45	59.41	5.62	0.52
NN: Shared words, TF-IDF normalization	11.37	86.04	1.81	0.77
NN: Shared words, n-grams	11.44	86.09	1.93	0.54
NN: Shared words, embeddings	36.01	57.12	6.23	0.64

© 2020 Parker ST. *JAMA Network Open.*

eTable 7. Comparison of National Estimates of Non-Fatal Shootings with NLP

	Unadjusted NEISS-FISS			NLP-Adjusted NEISS-FISS		
	Average Annual Estimate (No.)		95% CI	Average Annual Estimate (No.)		95% CI
Home	15,257	23.4	(8,543.9-21,969.5)	23,738.478	36.0	(14,335.2-34,656.5)
Street/Hwy	14,447	22.9	(5,525.5.4-23,368.9)	27,200.478	43.6	(13,769.6-45,517.5)
Other Public Place	7,732	11.6	(3,801.9-11,662.8)	10,439.261	17.9	(6,061.7-18,337.5)
Other	1,568	2.6	(803.9-2,332.2)	1,656.348	2.5	(913.2-2,474.8)
Unknown	29,029	39.5	(13,095.4-44,961.7)	0	0	
Total	68,033	100		63,035	100	

© 2020 Parker ST. *JAMA Network Open.*
